# Nelson's Syndrome: A Narrative Review

**DOI:** 10.7759/cureus.39114

**Published:** 2023-05-16

**Authors:** Jorge Alejandro Torres-Ríos, Gerardo Romero-Luna, Juan Marcos Meraz Soto, Lilian Zavala-Romero, Monica L Aguirre Maqueda, Alejandro Rodríguez Camacho, Sergio Moreno Jiménez

**Affiliations:** 1 Neurosurgery and Radiosurgery, National Institute of Neurology and Neurosurgery, Mexico City, MEX; 2 Radiosurgery, National Institute of Neurology and Neurosurgery, Mexico City, MEX; 3 Radioneurosurgery, XXI Century National Medical Center, Mexican Social Security Institute, Mexico City, MEX; 4 Neurology, ABC Medical Center, Mexico City, MEX

**Keywords:** cushing’s syndrome, pituitary adenoma, acth, cushing’s disease, nelson’s syndrome

## Abstract

Nelson's syndrome (NS) is an uncommon disease occurring as a complication of bilateral adrenalectomy (BLA) in patients with persistent Cushing's disease (CD) due to an adrenocorticotropin-producing pituitary tumor. The first reports of this syndrome were done in the 50s, although its pathophysiology is still not understood. Every year, between 1.8 and 2.6 cases are thought to occur per million people. It is characterized by hyperpigmentation, elevated adrenocorticotropic hormone (ACTH) plasma levels, and typical signs and symptoms related to pituitary adenomas, such as visual deficits due to optic pathway compression or decreased hormone production from the adenohypophysis. NS represents a challenge due to the lack of accepted diagnostic criteria and the complexity of its treatment. Moreover, the development of stereotactic radiosurgery (SRS) in the last few years has become an essential but controversial strategy for this syndrome. This review presents a comprehensive overview of NS.

## Introduction and background

Cushing's syndrome (CS) is characterized by elevated circulating cortisol levels. Clinical features of this condition include central fat, skin lesions, hypertension, hyperglycemia, osteoporosis, and vascular disease. Endogenous or exogenous features, such as prolonged steroid administration, can cause CS. On the other hand, Cushing's disease (CD) originated from the central nervous system, frequently from a functional pituitary adenoma that secretes adrenocorticotropic hormone (ACTH), which stimulates the adrenal glands and induces elevated levels of circulating cortisol. In 80% of cases, CD is the most frequent source of endogenous CS [1].

CD functional-related pituitary adenoma can be treated with transsphenoidal surgical resection. In most cases, biochemical remission and immediately circulating cortisol control result from the procedure, and patients may have a good prognosis [1,2]. Nevertheless, other treatment alternatives are available for unsuccessful surgical resection patients, such as surgical reintervention, medical therapy, radiotherapy, or bilateral adrenalectomy (BLA). The last is an ideal and effective therapy alternative for patients with hypercortisolemia due to refractory CD [2]. In addition, BLA can lead to complications such as hypercortisolism due to ACTH stimulation of residual adrenal tissue or the development of an aggressive ACTH-producing pituitary neuroendocrine tumor, a condition known as Nelson's syndrome (NS) [3].

NS was first described in 1958 by Dr. Don Nelson in a 33-year-old female patient diagnosed with CD and treated with surgical BLA. Three years later, this patient presented with skin hyperpigmentation, elevated ACTH plasma levels, and a pituitary tumor. These three symptoms then became the typical clinical triad of NS. In the 60s, Dr. Nelson and colleagues described a kind of ACTH-producing tumor that appeared in several patients after BLA, leading to elevated ACTH levels and hyperpigmentation. Since then, this disease has been called Nelson's syndrome [2,4].

Since the original description of this syndrome, physicians have been much concerned and trying to understand its pathophysiology and develop new treatments for refractory CD and NS. There is still no consensus on the NS definition currently. However, it can be defined as radiographic evidence of a pituitary tumor with increased plasma ACTH levels and skin hyperpigmentation following BLA due to CD [5,6].

Moreover, ACTH-secretory pituitary tumors have aggressive behavior and cause substantial morbidity. Treating this disease used to be complicated and, in most cases, required a multidisciplinary approach by surgical resection (transsphenoidal in most patients), stereotactic radiosurgery (SRS), and pharmacologic treatment [3].

NS is an uncommon complication of refractory CD treated with surgical BLA and is a rare diagnosis to consider when approaching adrenal-related diseases. 

## Review

Epidemiology

ACTH-dependent and ACTH-independent causes of CS can be distinguished. About 80% of adult instances of CS are caused by ACTH-dependent factors, and CD accounts for 85%-90% of these cases. Each year, between 1.8 and 2.6 cases of CD are thought to occur per million people; 4%-6% of pituitary adenomas are corticotrope adenomas, which are CD-related [7].

The prevalence of NS ranges between 5% and 53.4% with a history of BLA and positive imaging. Taking into count that NS is defined as a larger than 10% increase in tumor volume over the original tumor volume, the prevalence of NS ranges from 5% to 53.4% for positive imaging. For increased ACTH levels alone or in combination with skin pigmentation, the prevalence ranges from 32.4% to 55.6% [8-10]. Radiographic tumor growth, increased ACTH levels, and skin pigmentation range from 8.6% to 46.7%. When biochemical and clinical criteria were used for diagnosis, the median latency of NS development was one to six years. However, when just positive imaging was necessary, NS was detected at a mean period of two to three years after BLA. In trials where all requirements (radiographic, biochemical, and clinical) had to be satisfied, the mean time between BLA and NS diagnosis varied greatly, ranging from 15 months to 16 years [5,8-10].

Pathophysiology

**Figure 1:**
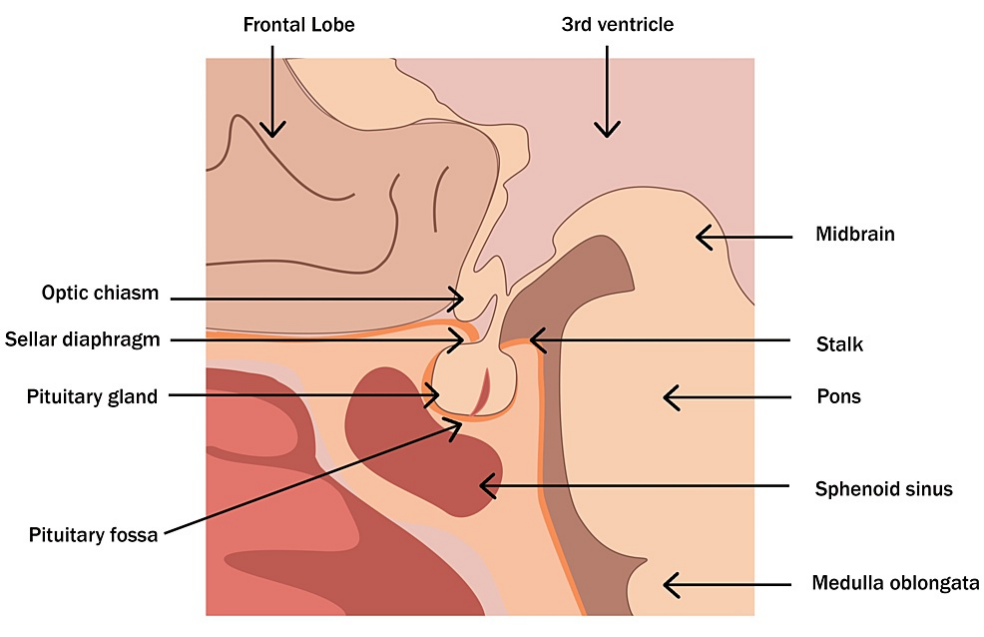
Pituitary gland The pituitary gland is located in the center of the skull base. The gland rests on the pituitary fossa and is separated from the rest of the skull by the sellar diaphragm, which has a central foramen that is crossed by the pituitary stalk. The latter is a cylindrical structure, which is formed mainly by axons that connect the posterior lobe of the pituitary gland with the hypothalamus. Image credit: The authors of the current study.

To understand NS pathophysiology, it is necessary to do a short review of the pituitary and sellar region anatomy and physiology. The pituitary gland is an essential element of the neuroendocrine system. This gland is five millimeters high, 10 millimeters deep, and 15 millimeters wide in adults. It is divided into two portions: the posterior portion, also known as neurohypophysis, which is in close contact with the hypothalamus and is in charge of oxytocin and antidiuretic hormone secretion; and the anterior portion, also known as adenohypophysis, which controls the production and secretion of various hormones [11]. It is located in the central nervous system, specifically in the sellar region, the brain’s center, and the skull base. The gland rests on the pituitary fossa and is separated from the rest of the skull by the sellar diaphragm. This meningeal folding forms a roof over the gland with a central foramen crossed by the pituitary stalk. The last is a cylindrical structure mainly made of axons that connect the posterior pituitary lobe to the hypothalamus (Figure 1) [12].

**Figure 2:**
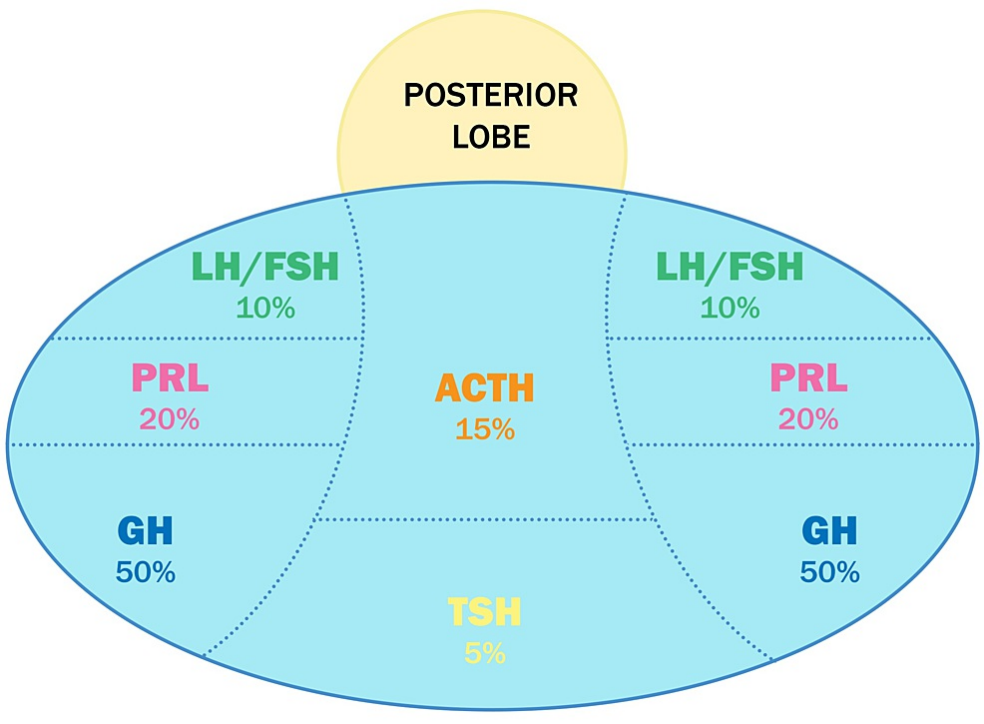
Distribution of cells in the pituitary gland In the anterior lobe of the pituitary, different types of cells are responsible for the manufacture of various hormones. About 20% of the anterior pituitary cells are lactotrophs that secrete prolactin (PRL), and about 50% are somatotroph cells that create growth hormone (GH). Corticotrophs account for 15% of the anterior pituitary cells and are in charge of producing adrenocorticotropic hormone (ACTH), and up to 10% are gonadotroph cells that generate luteinizing hormone (LH) and follicle-stimulating hormone (FSH). Thyrotrophs produce thyroid-stimulating hormone (TSH) and represent about 5% of the anterior pituitary, being the least prevalent cell type. Image credit: The authors of the current study.

The ACTH is one of the six hormones in the adenohypophysis that is involved in the hypothalamic-pituitary axis. This hormone stimulates the adrenal cortex, causing it to produce and secrete glucocorticoids. The ACTH is produced by corticotrophin cells, which represent 15% of pituitary cells and are in the center of the adenohypophysis (Figure 2) [13,14]. The corticotroph cells are regulated by the corticotropin-releasing hormone (CRH), which is produced in the paraventricular nucleus of the hypothalamus and governed by a feedback system repressed by glucocorticoids from adrenal glands that cross the blood-brain barrier to bind glucocorticoid nuclear receptor and inhibit CRH production at the paraventricular nucleus.

**Figure 3:**
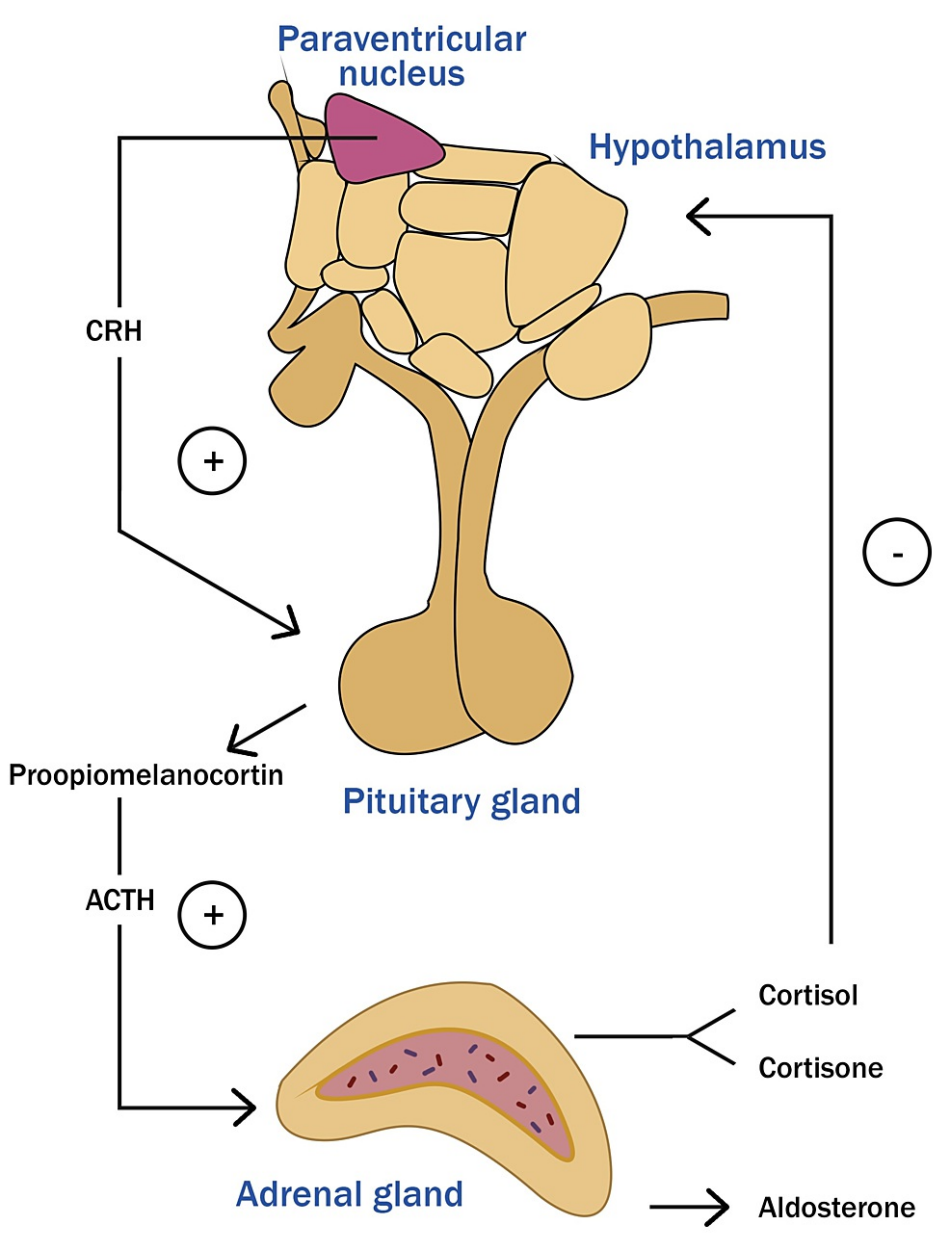
Hypothalamic-pituitary-adrenal axis Corticotroph cells are regulated by corticotropin-releasing hormone (CRH), produced in the paraventricular nucleus of the hypothalamus, whose activation is governed by a positive feedback system repressed by glucocorticoids from the adrenal glands. Corticotroph cells produce proopiomelanocortin, which is a precursor molecule of adrenocorticotropic hormone (ACTH) that is converted by proteolytic enzymes. Image credit: The authors of the current study.

The ACTH cells produce proopiomelanocortin, an ACTH precursor molecule converted by proteolytic enzymes into ACTH, and other molecules such as β-lipotropic hormone, melanocyte-stimulating hormone, and β-endorphin. Between 1 a.m. and 4 a.m., the levels of ACTH and cortisol start to increase, reaching their peak in the early morning. In the adrenal glands, ACTH stimulates the production of glucocorticoid and mineralocorticoid hormones. Glucocorticoids, such as cortisol and cortisone, are produced in the adrenal cortex and regulate proteins, lipids, and, especially, carbohydrates metabolism, promoting glucose rise through gluconeogenesis and glycogenolysis. Mineralocorticoids, such as aldosterone, are produced in the glomerular zone of the adrenal glands and regulate fluid and electrolyte balance, such as sodium, potassium, chlorine, and bicarbonate (Figure 3) [11,13-15].

**Figure 4:**
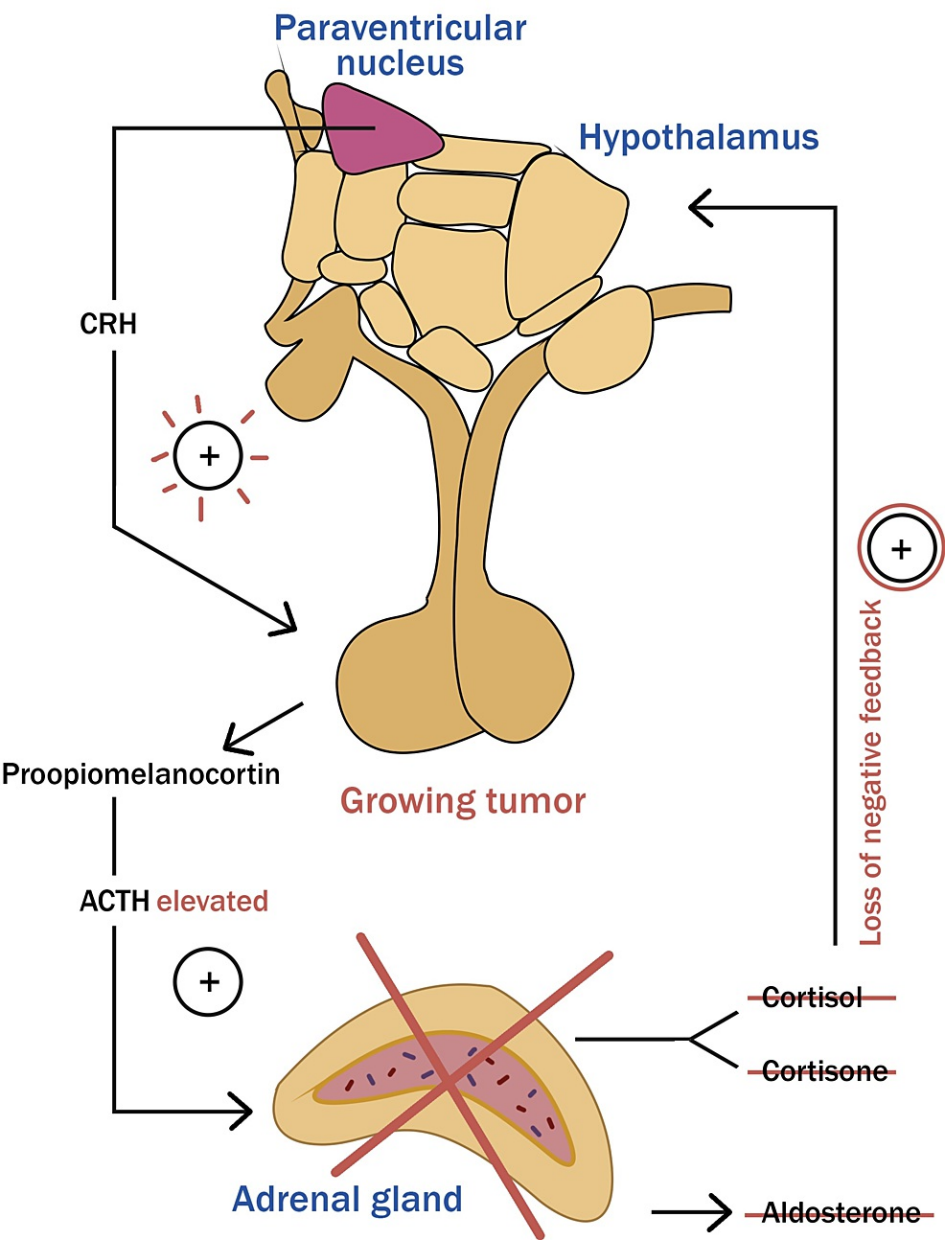
Hypothalamic-pituitary-adrenal axis altered in Nelson's syndrome Surgical resection of the adrenal gland is designed to reduce hypercortisolemia in patients with Cushing's disease (CD) by breaking the feedback loop. The tumor that eventually develops and gives rise to Nelson's syndrome is believed to originate from the corticotropic cells that caused CD. Image credit: The authors of the current study.

CS refers to the symptoms of hypercortisolism, regardless of the cause. The CD is used when hypercortisolism is caused by an ACTH-secreting pituitary adenoma, which accounts for 6% of all pituitary adenomas [16]. About 80% of T-PIT (T-box family member TBX19) lineage corticotropic tumors are microadenomas. Despite being uncommon, corticotroph macroadenomas are the second most common source of pituitary carcinomas [7]. Only a small percentage of CD cases, like multiple endocrine neoplasia 1 (MEN1), is caused by germline mutations that result in pituitary adenomas. The only treatment that can provide a permanent cure is the surgical excision of the pituitary adenoma. Preoperative medication adrenal blocking before surgery is not beneficial except for individuals in poor health [7]. With a mean of 75%-80%, postoperative remission rates ranged from 25% to 97%. Despite initial remission, 20% of patients experience CD recurrence within three to 345 months. Second-line therapies include BLA and pituitary irradiation [16]. The surgical resection of adrenal glands lowers hypercortisolemia in CD patients, breaking the system's negative feedback loop. The tumor that finally develops and results in the formation of NS is thought to have its origins in the corticotroph adenoma cells that cause the CD in the first place (Figure 4) [1-2,7,10].

NS, which might be considered an iatrogenic condition, typically develops one to four years after BLA. The first theory proposed that NS results from the loss of serum cortisol's ability to regulate the hypothalamus and pituitary gland after BLA because cortisol levels that previously suppressed the production of the CRH in the hypothalamus returned to normal, leading to an increase in the CRH production. Following BLA, there was an increase in hypothalamic transcription and a modest increase in corticotroph cells. The leftover tumor cells are stimulated to expand until they form a corticotroph adenoma and produce more proopiomelanocortin when CRH is elevated (Figure 4) [2,3,8].

The majority of corticotroph adenomas in NS patients produce more proopiomelanocortin transcription products. The ACTH reaction to CRH in these patients differs from that of patients with CD adenomas in two ways: it is more robust and lasts longer. In patients who have had BLA and have NS, the bigger tumor size and lower glucocorticoid feedback may be the causes of these disparities. Additionally, vasopressin V3 receptor genes are overexpressed in pituitary tumors that secrete ACTH, and both arginine vasopressin and CRH stimulate the growth of a corticotropic tumor cell line. The excess pigmentation exhibited in these patients is caused by proopiomelanocortin misfolding to ACTH and melanocyte-stimulating hormone. In an in vitro experiment, proopiomelanocortin-derived peptide secretion from a pituitary adenoma causing NS was reduced by somatostatin-14 and somatostatin-28. Still, it was enhanced by arginine vasopressin, vasoactive intestinal peptide, and oxytocin. Plasma ACTH levels in the NS are stable and exhibit only slight diurnal variation, demonstrating that this mechanism is not dependent on physically released CRH. In patients with NS compared to patients with CD, basal ACTH secretion is raised by a factor of six and pulsatile secretion by a factor of nine [3-5,8].

All patients with total BLA are given steroid replacement therapy, which typically amounts to daily doses of 30 milligrams of hydrocortisone. The maintenance dose and frequency of glucocorticoid treatment may partially explain the various occurrences of NS following BLA for CD. The ACTH-producing pituitary cells may become overstimulated when the inhibitory effect of steroids is insufficient for extended periods as in situations where glucocorticoid hormone replacement has been inadequate. This may cause these cells to become adenomatous and transform into NS [5,8].

The absence of sensitivity of adrenocorticotropic cells to steroid hormone replacement therapy remains intriguing in the etiology of NS. Similar to corticotroph adenomas, NS adenomas have a suboptimal response or partial resistance to glucocorticoids. Numerous studies demonstrate that glucocorticoid hormone therapy does not often reduce plasma ACTH levels in individuals with NS. The carcinogenesis of the ACTH pituitary adenoma with this mutation has been hypothesized to include a structural flaw in the glucocorticoid receptor [3,8].

It is well accepted that the fundamental etiology of ACTH-secreting pituitary adenomas is a failure of the cortisol feedback system following BLA. Another less widely accepted theory holds that the aggressiveness of these adenomas was preprogrammed. It is now believed that the corticotroph adenoma in the NS most likely arises from the original tumor cell population responsible for CD, despite the fact that NS tumors typically tend to be larger, more invasive macroadenomas [8]. E-cadherin participates in cell-cell adhesion in epithelial tissues, and studies have linked decreased expression of this protein to higher tumor invasiveness and metastatic potential. Compared to corticotroph microadenomas or macroadenomas, which are corticotroph tumors, NS tumors have the least expression. Additionally, the frequency of USP8 mutations in NS tumors and CD was comparable, demonstrating that USP8 mutations are not the cause of the development of corticotroph adenoma in NS (Figure 4) [5].

Clinical features

Given the widespread expression of its receptors, chronic hypercortisolism has numerous effects on the entire body. Clinical indications of CS could include generalized growing symptoms such as obesity, hypertension, and irregular menstruation [17]. Ecchymoses, plethora, proximal muscular weakness, osteopenia/osteoporosis, and wide purple striae are other less typical but more distinct signs of CS. Fractures frequently contrast with average or little altered bone mineral density in glucocorticoid-induced osteoporosis. Children's growth slowed down as well [7,17].

Most patients go through emotional and cognitive changes, which often include increased tiredness, irritability, depression, sleeplessness, and poor memory and attention. The first signs frequently show irritability and sleep difficulties with early morning awakenings. In contrast to the persistent distress of depressed individuals without CS, the time history of depression symptoms is characterized by alternation with intervals of increased arousal [18,19].

Additionally, as cortisol can bind to the mineralocorticoid receptor in the kidney and brain, fluid and electrolyte problems can be explained. Cortisol is converted by the enzyme 11-hydroxysteroid dehydrogenase type 2 (11BHSD2) into the inactive molecule cortisone, which precludes it from being a significant physiological mineralocorticoid [20]. When cortisol has raised to levels that override the ability of 11BHSD2 to keep it inactive, cortisol exerts aldosterone-like effects in the kidney, which is why when cortisol is pathologically high, salt retention, potassium squandering, and hypertension can ensue [16].

Catabolic symptoms exist now in many people who did not previously have them. However, screening for these alterations is ineffective, and people with diabetes and/or systemic hypertension do not exhibit them.

The leading cause of NS-related complications is tumor development. The main clinical characteristics seen are related to pituitary hormone deficiency. Any anterior pituitary hormone can be blocked from being released, and tumor compression occasionally results in diabetes insipidus. A significant tumor can impair vision through compression of the optic pathways; this impairing of vision has been documented in 10%-57% of instances. By affecting the oculomotor, trochlear, abducent, and V1 and V2 branches of the trigeminal nerve, a tumor that invades the cavernous sinus can result in diplopia and cranial nerve abnormalities. A tumor must be large enough to restrict the passage of cerebrospinal fluid (CSF) for features of elevated intracranial pressure to manifest. These features are rare and can develop later. The diaphragm sellae may be stretched, which can result in headaches. There have been cases of malignant transformation into pituitary cancer with distant metastases [6,8,21].

Skin hyperpigmentation is typically noticeable, not just in places exposed to the sun. It varies based on ethnicity and melanocyte-stimulating hormone concentrations. The linea nigra, pigmentation of the extensor surfaces, scars, gingivae, and areolae are typical symptoms of hyperpigmentation in patients [8,16,21].

Adrenal cortical cells may move down the line of gonadal descent or become trapped in the testicular hilum during development, giving rise to adrenal rest tissue. This resting adrenal tissue may get activated in NS. When in the testes, it may cause oligospermia and painful testicular enlargement. Rarely, the adrenal rest tissue may produce enough cortisol to restore the levels or even trigger CD to return [6,8].

Diagnosis

There is no fully accepted consensus for the diagnostic criteria of NS. The current diagnostic standards for NS center on skin hyperpigmentation, an increasing pituitary tumor post-BLA, and rising plasma ACTH levels. A remnant’s presence is insufficient evidence of pituitary tumor growth [8,22]. Skin pigmentation intensity and ACTH levels are unrelated; however, some individuals may only experience it when their ACTH levels are exceedingly high, which raises sensitivity concerns [23].

NS diagnosis includes pituitary residual tumor regrowth, and magnetic resonance imaging (MRI) can detect the tumor regrowth early on before it develops into a macroadenoma. Barber et al. [6] proposed the following diagnostic criteria for the diagnosis of NS. A patient must meet at least one of the following two requirements in addition to having previously undergone complete BLA for CD to be diagnosed with NS: (1) an increasing mass lesion in the pituitary after BLA surgery, as shown on an MRI or CT scan, as opposed to an MRI of the pituitary before BLA surgery and (2) an elevated level of ACTH to >500 ng/L from a single plasma sample taken at 08:00 h before steroid administration and post-BLA surgery as well as progressive elevations of ACTH levels from plasma samples taken on at least three consecutive occasions at different time-points post-BLA surgery or an increase of ACTH by 30% of the initial post-BLA sample [8,22,24].

NS can be diagnosed if at least one of the two following conditions is met: (1) an elevated level of ACTH >500 ng/L from a single plasma sample in addition to progressive elevations of ACTH levels from plasma samples taken on at least three consecutive occasions at different time-points post-BLA surgery or (2) an expanding pituitary mass lesion post-BLA surgery compared with MRI of the pituitary before BLA surgery [5-6,10].

Predictive factors

Due to its link to tumor growth, high ACTH levels in the first year after BLA are the most reliable prognostic predictor. An ACTH cut-off value of more than 100 pg/mL increase in the first year following BLA has been proposed [22] as a predictor of corticotroph tumor progression.

The presence of pituitary adenoma before BLA has also been considered a risk factor. However, this is yet to be proven [10,22]. It has been proposed that prophylactic pituitary irradiation administered concurrently with BLA may play a preventive effect in lowering the likelihood of NS development or even postponing its onset [2,5,25].

NS has been linked to a residual pituitary adenoma before BLA. NS has been demonstrated to be predicted by the presence of an adenoma during surgery or on neuroimages, especially in patients with more extensive tumors, specifically macroadenomas with cavernous sinus involvement [2,25].

Although it has been hypothesized that inadequate glucocorticoid replacement therapy following BLA, such as lack of steroids or insufficient low doses, may contribute to the development of NS, further research has not been done to evaluate this component [26].

Surgical treatment

NS must be treated using a multidisciplinary strategy. Medical care, radiation, and surgical pituitary removal are the mainstays of treatment.

The first line of treatment for reducing tumor size and secretion is surgical resection. Although transcranial surgery can occur if the extracellular extension is present, a transsphenoidal method is more frequently used [3,27]. During varying follow-up intervals, surgical removal of NS tumors has been linked to 0% and 50% growth rates.

The surgical approach depends on the characteristics of each patient. The transsphenoidal approach is the best option for those patients with microadenomas or macroadenomas without invasion. On the other hand, the transcranial approach is recommended in patients coursing with tumors with extrasellar or parasellar invasion. A combined approach can be carried out in the case of a giant tumor. Because of a recurrence tendency and disappointing results in patients treated with selective adenectomy, total hypophysectomy was the selection procedure [28,29].

Overall, the success rates for surgical treatment of NS range from 10% to 70%. Early neurosurgical therapy of tiny tumors before extra-sellar involvement is noticed to have a higher likelihood of producing favorable results [3,30]. Success in the surgical treatment of NS is primarily defined by long-term remission and minimal complication [30]. The 10-year tumor progression-free survival is 80% for patients treated with surgery [31]. The criteria for NS remission after treatment are as follows: (1) ACTH < 200 pg/mL two hours after the morning dosage of glucocorticoids, (2) tumor size less than 10 mm and ACTH less than 200 pmol/L following the cessation of glucocorticoid replacement therapy for at least 24 hours, (3) ACTH less than < 70 pg/mL two hours following the morning glucocorticoid dose and no signs of tumor development, and (4) ACTH less than 200 pg/mL after 24 hours of no glucocorticoid replacement medication and no tumor on MRI [5,31].

The reported remission rates ranged from 33% to 100% [5,31]. Panhypopituitarism (69%), chronic diabetes insipidus (38%), cerebrospinal fluid leak (15%), meningitis (8%), cranial nerve palsy (5%), and death (5%) are some of the adverse effects. Although surgery is the first line of treatment for NS, it is less successful in cases where extrasellar involvement is present. Radiation therapy would be appropriate for these people [3].

Radiosurgery and radiotherapy

Stereotactic fractionated radiation therapy (SFRT) is used in the care of NS to prevent tumor growth, decrease plasma ACTH, and manage hyperpigmentation due to the low cure rates of surgical surgery alone. Following surgical removal of pituitary tumors and BLA, SFRT achieves effective management rates of up to 90% for tumor growth control and 70% for ACTH level reduction [5,32].

According to current data, SRS is the most frequent radiosurgical procedure performed after a diagnosis of neuropathy, which consistently controls tumor growth in 90% of patients and results in successful ACTH decreases in 67%-100% of patients [5]. However, in up to 17% of patients, SRS may normalize serum ACTH. In 7%-40% of patients, new-onset pituitary hormone shortage, temporary partial III and VI cranial nerve, and permanent nerve or visual abnormalities are the expected consequences of SRS [5].

When SRS is used in patients with NS, the goal is to treat all visible tumor tissues with an adequate margin. The prescribed dose must be discussed for each patient. The target is to cover the volume with a minimal isodose of 50% with 15 Gy. Prescribed doses can be raised to 25 Gy if risk organs, such as the optic pathway and pituitary stalk, are safe. In cases where the tumor margin is less than 2 mm from the visual pathway, a hypofractionation of the total dose can be carried out in three fractions for three consecutive days, looking to provide a full dose of less than 8-10 Gy to the optic pathway [33].

In the Losa et al. series, the progression-free survival at 10 years is 91%, with a median volume reduction of 24.8% and a median plasma ACTH level reduction of 78% in patients who received SRS. These values vary in the literature according to the follow-up time. It is essential to mention that follow-up is obligatory for all patients for at least five years, although the risk of recurrence stays forever [32].

Prophylactic pituitary SFRT has also been used to reduce NS risk after BLA and has shown an incidence reduction in some series [34]. However, other studies have shown an increased risk. There still needs to be more data to determine the role of radiation therapy, fractionated or SRS, in preventing NS. It is advised to avoid its routine use and to make an interdisciplinary discussion of its utility in patients with invasive macroadenomas with subtotal resections [28].

Complications in that study were limited to new pituitary hormone deficiency (10%), with no patients developing cranial neuropathy or visual deficits. The risk of developing NS in patients who do not receive prophylactic SFRT is up to 50% [25].

Medical treatment

While the cornerstone of NS treatment is based on surgical resection of the pituitary neuroendocrine tumors (PitNET), medical treatment is also an important part of the approach for patients with this disease.

The treatment of aggressive pituitary tumors with chemotherapy has always been with temozolomide (TMZ), which was described for the first time in 2006 with partial success. This drug exerts its cytotoxic activity by alkylating DNA at the O^6^-methylguanine methyltransferase (MGMT) position of guanine, resulting in irreversible DNA damage [35]. Clinically functional tumors have shown an essential improvement in the symptoms and tumor size reduction when treated with SFRT associated with TMZ. Ji et al. reported a systematic review including 31 cases of aggressive pituitary adenomas treated with TMZ, which stated that 48.4% had partial/complete response, 29% had stable disease, and 22.5% had a lack of response when treated only with TMZ, arguing that most patients responded well to the chemotherapy treatment. However, it should be studied with other chemotherapeutic drugs for better outcomes [35].

Treatment with TMZ combined with capecitabine (CAP) has also been studied, but it still needs to be determined due to the lack of evidence. CAP is metabolized to 5-fluorouracil, affecting DNA synthesis and replication. It has significant adverse effects such as bone marrow suppression, diarrhea, hand-foot syndrome, nausea, or fatigue.

Zacharia et al. demonstrated in a four-patient case series that two patients showed complete tumor regression, one had a 75% shrinkage of the adenoma, and the last one had 4.5 years with a stable disease that progressed at last [36]. This indicates that it can be an available treatment with a low toxicity rate for this pathology. However, only some successful cases have been reported in the literature, and every patient should be first screened for hematologic diseases due to the adverse effects of CAP. Also, it causes much fatigue in the patients, worsening their clinical presentation and referring them to another treatment rather than feeling tired most of the time, despite the fact that the best treatment should be the least harmful, most effective, and best tolerated [37,38].

Sodium valproate, cyproheptadine, and bromocriptine have also been shown to reduce ACTH serum levels. In some cases, they reduced tumor size, but long-term treatment has shown a negative effect on this pathology, causing the progression of the symptoms and having several side effects [39].

There is not enough evidence of medical treatment for this disease; it is still recommended that TMZ should be used individually in macroadenomas that cannot be treated with radiation therapy or surgery [28].

## Conclusions

NS is one of the most challenging endocrine conditions. Although its mortality rate has decreased over the years, it remains associated with significant morbidity. Since this complication often has an aggressive presentation, screening is necessary to diagnose patients with NS early. Standardizing diagnostic criteria to facilitate its approach and homogenize information is necessary. It is essential to emphasize that there is no standard treatment for all patients with NS; it requires a multidisciplinary approach where neurosurgeons, neurologists, endocrinologists, surgeons, radiation oncologists, and internists play an essential role in aiming for the best prognosis and life quality for the patient.
